# Strategies for rapid reconstruction in 3D MRI with radial data acquisition: 3D fast Fourier transform vs two-step 2D filtered back-projection

**DOI:** 10.1038/s41598-020-70698-4

**Published:** 2020-08-14

**Authors:** Jinil Park, Jeongtaek Lee, Joonyeol Lee, Seung-Kyun Lee, Jang-Yeon Park

**Affiliations:** 1 Department of Biomedical Engineering, Sungkyunkwan University, Suwon, South Korea; 2 Center for Neuroscience Imaging Research, Institute for Basic Science (IBS), Suwon, South Korea

**Keywords:** Biomedical engineering, Applied physics

## Abstract

For 3D radial data reconstruction in magnetic resonance imaging (MRI), fast Fourier transform via gridding (*g*FFT) is widely used for its fast processing and flexibility. In comparison, conventional 3D filtered back projection (*c*FBP), while more robust against common radial *k*-space centering errors, suffers from long computation times and is less frequently used. In this study, we revisit another back-projection reconstruction strategy, namely two-step 2D filtered back-projection (*ts*FBP), as an alternative 3D radial MRI reconstruction method that combines computational efficiency and certain error tolerance. In order to compare the three methods (*g*FFT, *c*FBP, and *ts*FBP), theoretical analysis was performed to evaluate the number of computational steps involved in each method. Actual reconstruction times were also measured and compared using 3D radial-MRI data of a phantom and a human brain. Additionally, the sensitivity of *ts*FBP to artifacts caused by radial *k*-space centering errors was compared with the other methods. Compared to *c*FBP, *ts*FBP dramatically improved the reconstruction speed while retaining the benefit of tolerance to the radial *k*-space errors. Our study therefore suggests that *ts*FBP can be a promising alternative to the conventional back projection method for 3D radial MRI reconstruction.

## Introduction

Radial acquisition (RA), also known as projection acquisition, embodied the first *k*-space trajectory used in MRI, as proposed by Lauterbur^1^. Here the *k*-space is filled with a set of radial spokes that extend out from the origin. The RA scheme has recently gained popularity in the MR community due to the possibility of a shorter minimum echo time (TE) and better tolerance to motion and flow artifacts^2–4^ as compared to conventional Cartesian *k*-space acquisition. It is, however, sensitive to system imperfections such as timing delays between the actual and requested starting points of gradient waveforms^5,6^ because the readout direction in the *k*-space varies between repetitions. Delays and other forms of imperfections in the gradient system for radial sampling can cause the spokes to miss the center of the *k*-space, possibly resulting in severe artifacts in the final image. To correct the gradient-delay effects in RA imaging, several methods have been proposed to estimate and compensate for them^5–7^. The RA scheme also computationally burdens the image reconstruction system more than the Cartesian *k*-space acquisition scheme because the data must lie on a Cartesian grid in order to take advantage of the high computational speed afforded by the fast Fourier Transform (FFT) algorithm.

In terms of image reconstruction approaches, radial data can be reconstructed by two categories of reconstruction methods: 1) FFT via gridding (*g*FFT) and 2) filtered back-projection (FBP) based on the Radon transform^8^. The latter exploits the Fourier slice theorem^9^, which states that the Fourier transform of spoke-encoded data is the projection of an object perpendicular to the spoke direction. A major difference between *g*FFT and FBP is that the former performs image reconstruction in the *k*-space while the latter does so in the image space. Although both methods have been successfully used, *g*FFT is often preferred to the FBP approaches for 3D radial-data reconstruction due to its much shorter processing time and flexibility to handle irregular *k*-space patterns. For conventional 3D FBP (*c*FBP), a set of planar projections are obtained using the 3D projection slice theorem and then back-projected onto the 3D image space for reconstruction. The most time-consuming part of *c*FBP is the 3D volumetric back-projection of every radial spoke, which makes the computational time grow rapidly with the image resolution. Lauterbur and Lai proposed a much faster alternative to this straightforward but time-consuming *c*FBP method in 1980^10,11^. This approach, which will hereafter be called “two-step 2D FBP” (*ts*FBP), has been used in X-ray imaging but has received little attention from the MR community. Considering the growing role of 3D radial scans in MRI, we believe that it is timely to systematically compare the computational speed and error tolerance of the different radial reconstruction methods.

A main goal of this study is to evaluate the computational performance of three different 3D radial-scan reconstruction methods: *g*FFT, *c*FBP, and *ts*FBP. Mathematical expressions for the computational requirements of each reconstruction method were formulated by calculating the number of major operations in the reconstruction process. Experiments were also performed to demonstrate the robustness of the FBP approaches against simulated radial off-centering errors of the *k*-space trajectory, that can be caused by gradient delays, eddy currents, or finite digitization bandwidth, in comparison to *g*FFT. Recently it has been shown that *g*FFT can be further accelerated by utilizing algorithms tailored to specific radial trajectories^12^. In the present work, we considered only the most widely used, conventional gridding algorithm in *g*FFT for simplicity.

## Results

### Simulations

**Figure 1:**
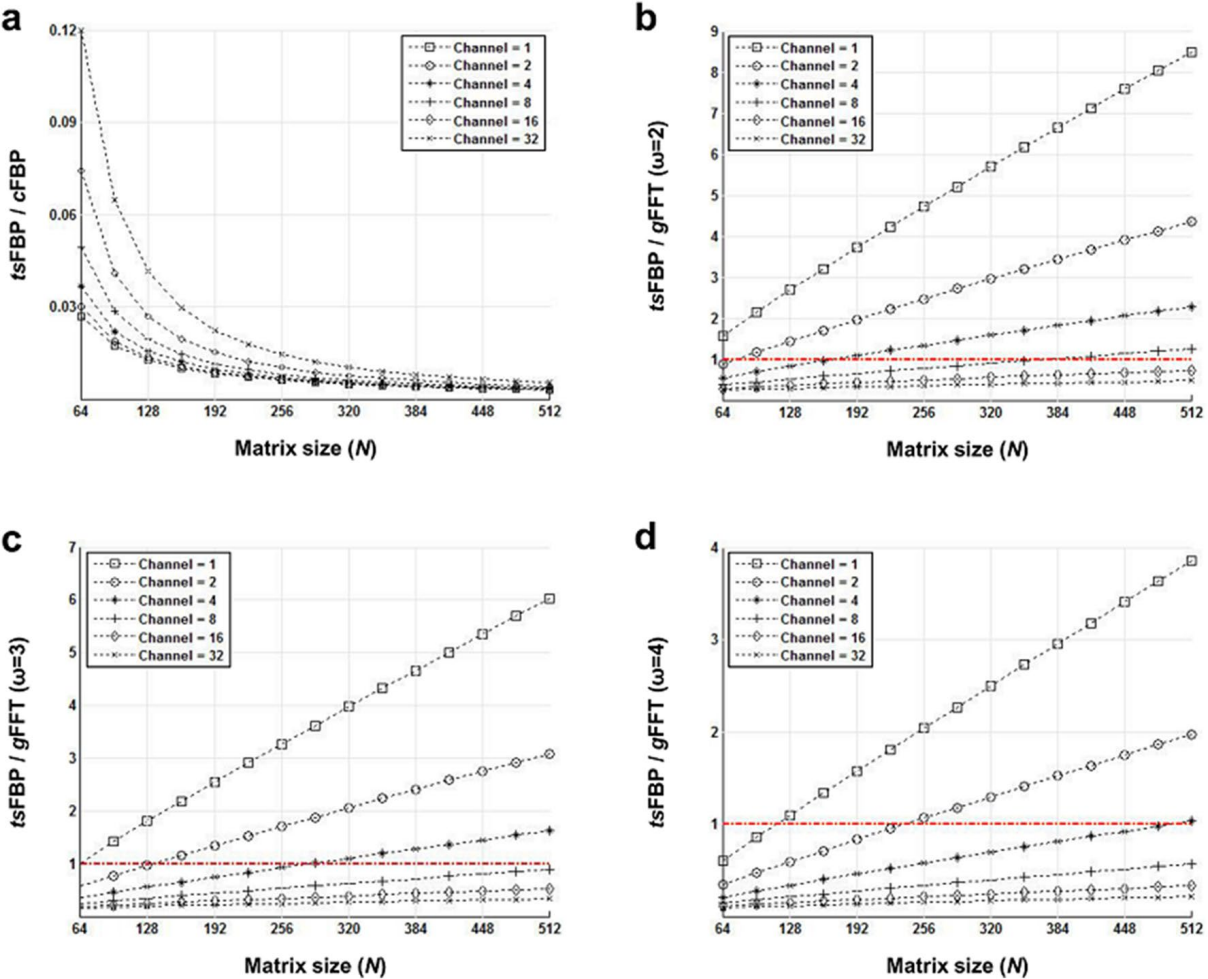
Comparison of the number of main computational operations between two-step 2D FBP (*ts*FBP) and conventional 3D FBP (*c*FBP) (**a**), as well as between *ts*FBP and 3D FFT via gridding (*g*FFT) (**b**–**d**). The ratios of operational counts between different methods are plotted as a function of the final matrix size (*N*) with a varying number of channels. The final image matrix was assumed to be cubic (*N* = *N*_x_ = *N*_y_ = *N*_z_). The oversampling factor was two in all cases, and the convolution-kernel width was two (**b**), three (**c**), and four (**d**). The dash-dot red lines indicate a threshold at which *ts*FBP and *g*FFT have the same computational burden.

Figure 1 shows the ratios between the operational counts (Eqs. 4–6) of *ts*FBP and the other two methods for varying numbers of receiver channels and image matrix sizes. *ts*FBP showed a dramatic decrease in computational requirement in comparison to *c*FBP for all cases (Fig. 1a). Under the channel combination scenario considered (see “Methods”), *ts*FBP also showed computational speed advantage over *g*FFT in cases where the matrix size (*N*) was small, or the number of channels (*N*_Ch_) was large (Fig. 1b–d). For example, when the convolution-kernel width for gridding (*ω*) was two, which was the case for our *g*FFT reconstruction of the real data, *ts*FBP outperformed *g*FFT when *N*_Ch_ ≥ 16. On the other hand, when *N*_Ch_ = 1, which corresponds to another likely scenario of channel combination (see “Discussion”), *ts*FBP took more computational steps than *g*FFT (by up to × 9) except with the largest *ω*. Under this scenario, *ts*FBP still had a large advantage over *c*FBP, by a factor of > 30.

Table 1 summarizes the theoretically estimated computational burdens of the three reconstruction methods and the actual computation times measured during the real data reconstruction using the same acquisition and reconstruction parameters. While the theoretical estimations of *c*FBP and *g*FFT were ~ 220 and ~3 times higher than those of *ts*FBP, respectively, their actual computational times were ~ 253 and ~ 8 times higher for the acquisition parameters used in the 3 T experiment. With the 9.4 T acquisition setup, the actual reconstruction times of *c*FBP and *g*FFT were longer than *ts*FBP by factors of ~ 143 and ~ 0.8, respectively, while the factors were ~150 and ~ 0.5 in theoretical calculations. The agreement between the actual computational times and the theoretical expectations appears reasonable given the approximate nature of the theoretical estimation.

**Table 1:** Mathematical expressions for the number of main operations and estimated computational burdens for specific acquisition/reconstruction parameters.

Methods	Computation processes	Number of main operations^a^	Estimated computational burden		Actual computation time	
			3 T^b^	9.4T^c^	3 T^b^	9.4T^c^
Two-step 2D FBP	1D FFT	*N*_v_(*N*_s_log_2_(*N*_s_))*N*_Ch_	6.3 × 10^10^	7.3 × 10^9^	1 min 30 s	40 s
	1^st^-step 2D FBP	(*N*_v,θ_*N*_x_*N*_z_ )*N*_v,φ_				
	2^nd^-step 2D FBP	(*N*_v,φ_*N*_x_*N*_y_)*N*_z_				
3D FFT via Gridding	Gridding	*N*_v_*N*_s_*ω*^3^*N*_Ch_	2.1 × 10^11^	3.7 × 10^9^	10 min	30 s
	3D FFT	*V*^3^*N*_x_*N*_y_*N*_z_log_2_(*V*^3^*N*_x_*N*_y_*N*_z_)*N*_Ch_				
	Intensity Correction	*N*_x_*N*_y_*N*_z_*N*_Ch_				
Conventional 3D FBP	1D FFT	*N*_v_(*N*_s_log_2_(*N*_s_))*N*_Ch_	1.4 × 10^13^	1.1 × 10^12^	380 min	95 min
	3D FBP	(*N*_x_*N*_y_*N*_z_)*N*_v_				

The actual computation times for the same parameters are also listed for comparison.

^a^For details, see “Methods”.

^b^*N*_v_ = 64,800, *N*_s_ = 504, *N*_v,φ_ = 180, *N*_v,θ_ = 360, *N*_x_ = *N*_y_ = *N*_z_ = 600, *V* = 2, *ω* = 2, *N*_Ch_ = 4.

^c^*N*_v_ = 64,800, *N*_s_ = 128, *N*_v,φ_ = 180, *N*_v,θ_ = 360, *N*_x_ = *N*_y_ = *N*_z_ = 256, *V* = 2, *ω* = 2, *N*_Ch_ = 1.

### Experiments

**Figure 2:**
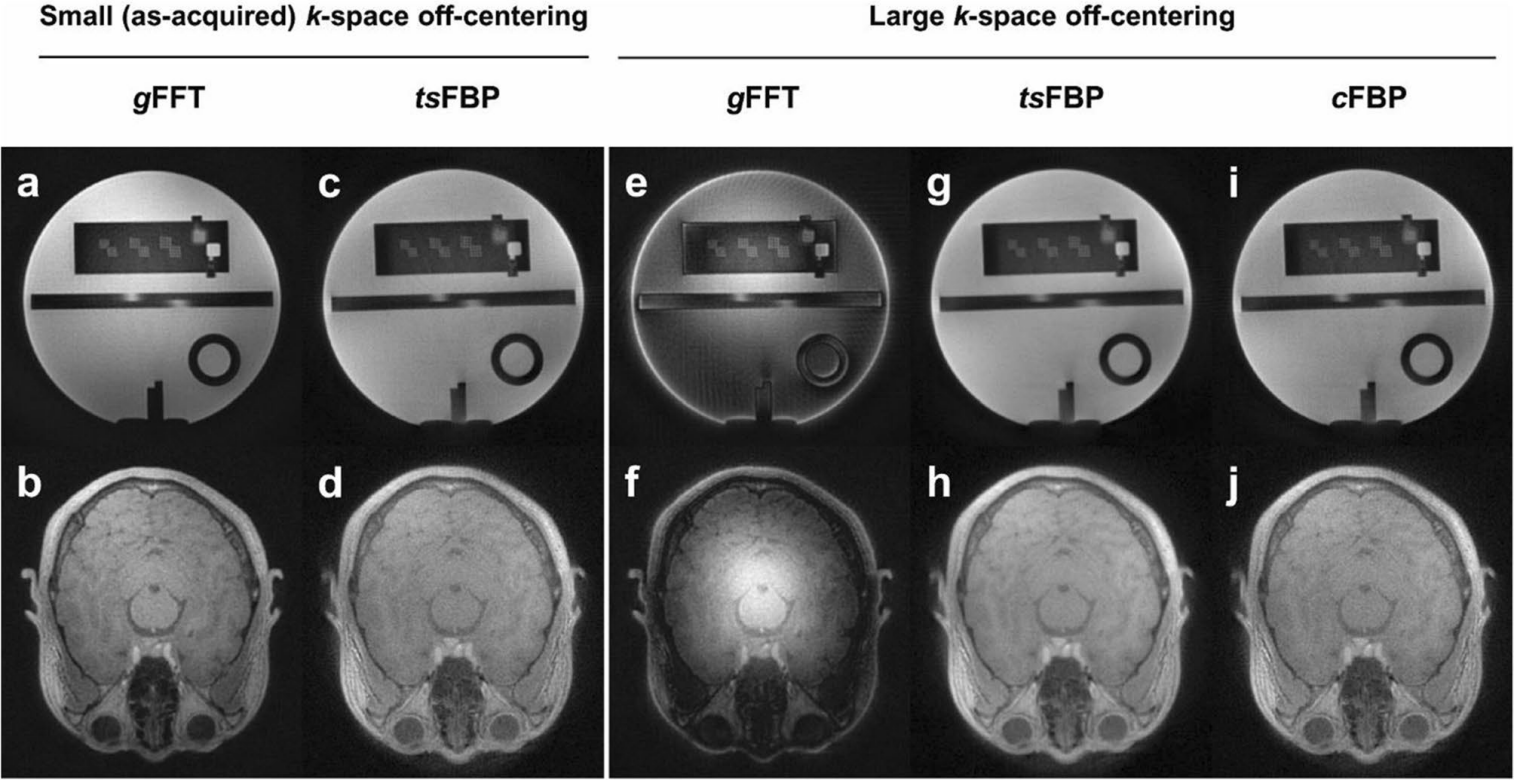
Axial slices of the ACR phantom and human brain images for two different degrees of *k*-space off-centering. (**a**–**d**) Images reconstructed from as-acquired raw data with small *k*-space off-centering from system imperfections. (**e**–**j**) Images reconstructed from the artificially echo-peak-shifted data.

Figure 2 shows the representative axial images of the American College of Radiology (ACR) phantom and the human brain reconstructed by *g*FFT, *ts*FBP and *c*FBP. Image qualities obtained from *ts*FBP and *g*FFT were similar when the off-centering of the *k*-space trajectory was small (Fig. 2a–d). However, in the presence of large *k*-space off-centering due to artificial echo shifts, *g*FFT showed contrast-varying artifacts from center to periphery (Fig. 2e–f). This is because *g*FFT adds an echo-shift-dependent phase modulation to the final image, resulting in undesired contrast variations, whereas *ts*FBP and *c*FBP are insensitive to phase-related artifacts thanks to magnitude-based projections. For detailed information of the echo-peak distributions of both as-acquired and echo-peak-shifted data, refer to Supplementary Fig. S1.

**Figure 3:**
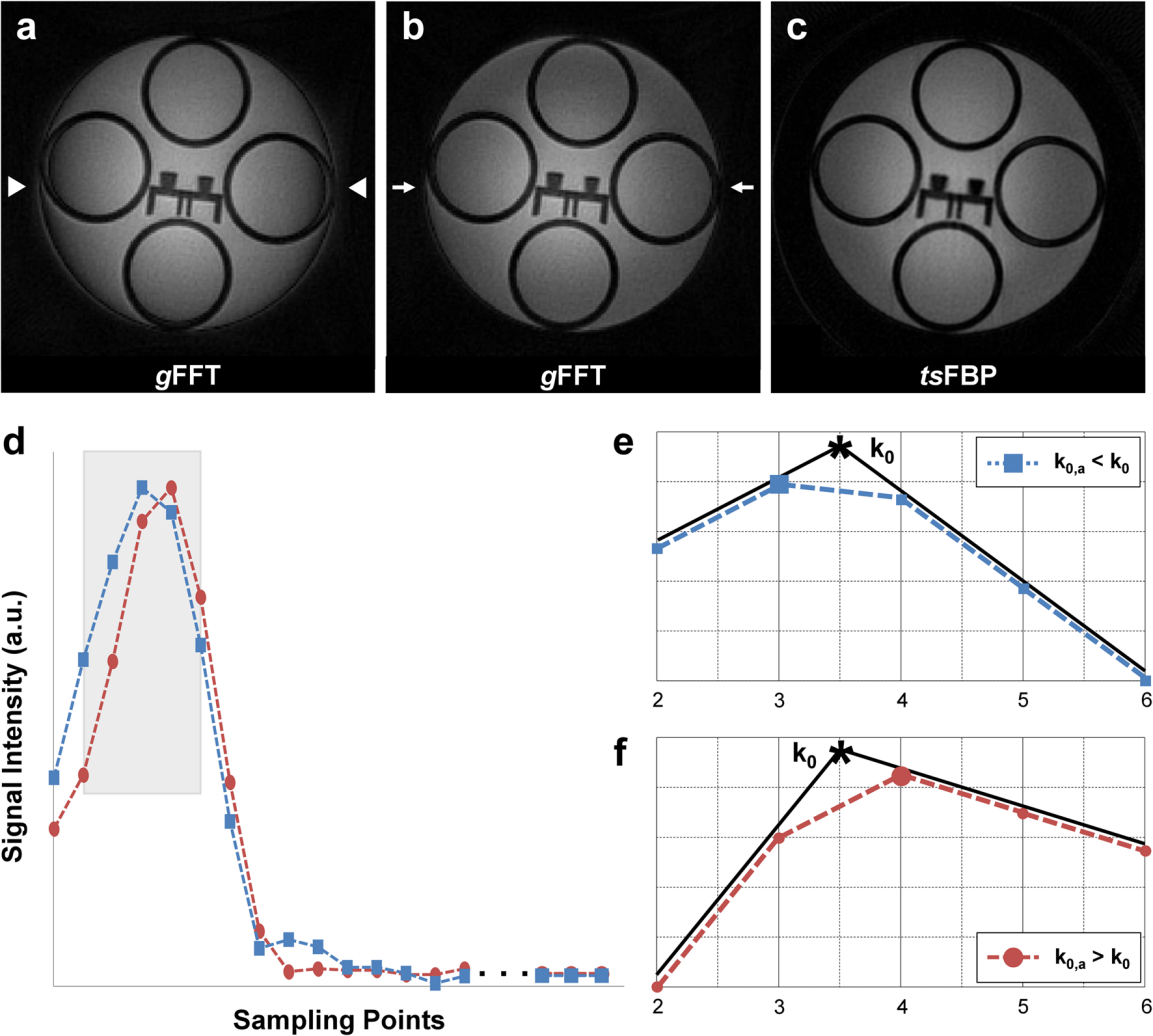
Reconstructed axial slices of the phantom at 9.4 T and illustration of peak positions of partial gradient echo signals. (**a**) Image reconstructed with *g*FFT using acquired raw data showing contrast-varying artifacts (white arrowheads). (**b**) Improved image (white arrows) reconstructed with all of the partial gradient echoes shifted by *k* = 0.5 point. (**c**) Image reconstructed with *ts*FBP without shifting of the partial gradient echoes. (**d**) Illustration of an example of two *apparent k* = 0 positions (*k*_0,a_), neither of which exactly corresponds to the *true k* = 0 (*k*_0_) due to the finite sampling bandwidth. The blue-dashed line with squares (**e**) and red-dashed line with circles (**f**) indicate a peak shift by one-half of a sampling interval to the left and to the right, respectively, compared to the *true k* = 0 (black asterisk marker).

Figure 3 shows another potential advantage of FBP over *g*FFT in terms of defining the “*true k* = 0” in discrete sampling. As shown in Fig. 3a a ring-shaped contrast variation is seen at the periphery of the phantom (white arrowheads) when a mismatch between the *true k* = 0 and the *apparent k* = 0 exists in *g*FFT. Figure 3d illustrates an example of two *apparent k* = 0 positions (*k*_0,a_), neither of which exactly corresponds to the *true k* = 0 (*k*_0_). Due to the finite sampling bandwidth, *k*_0,a_ could be located to the left (*k*_0,a_ < *k*_0_; blue-dashed line), or to the right (*k*_0,a_ > *k*_0_; red-dashed line) of *k*_0_, as shown in the detailed views of Fig. 3e,f, respectively. In our data, the edge artifact for *g*FFT nearly disappeared (white arrows) when *k*_0,a_ was shifted by a half of the sampling interval, which implies that such a slight shift could better match *k*_0,a_ to the true *k* = 0. In contrast, the image reconstructed by *ts*FBP showed little boundary artifact without any shift (Fig. 3c) since *ts*FBP exploits the magnitude-based projections for reconstruction and is thus insensitive to the phase-related errors as mentioned above. More examples including the human brain as well as the phantom images, which were reconstructed with *g*FFT while varying the *apparent k* = 0 position during the reconstruction, are presented in Supplementary Figs. S2–S4.

**Figure 4:**
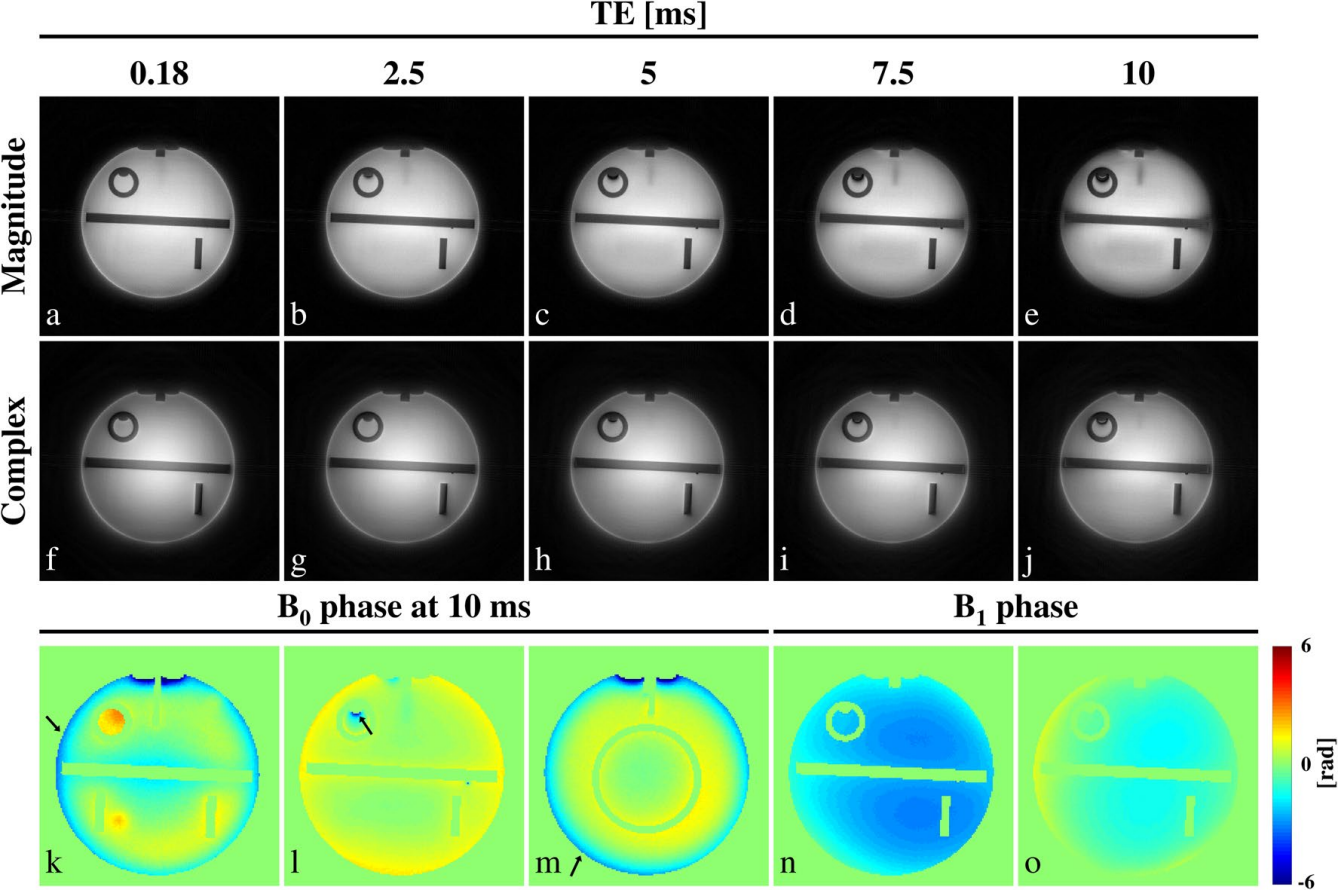
ACR phantom images (**a**–**j**) and phase maps (**k**–**o**). The images were acquired at 3 T at 5 different echo times using magnitude (**a**–**e**) and complex (**f**–**j**) projections. The phase maps were calculated from gradient echo phase images with the same echo times. The maps (**k**–**m**) correspond to the phase due to B_0_ inhomogeneity at TE = 10 ms on slices z = − 78.4 mm, − 22.4 mm, 73.6 mm, respectively, relative to the slice iso-center. The phase maps (**n**,**o**) represent the RF phase for the two channels of the coil, obtained by extrapolating each voxel’s TE-dependent phase to TE = 0. Scan parameters are TE = 0.18/2.5/5.0/7.5/10 ms, TR = 13 ms, FA = 5°, FOV = 300 × 300 × 300 mm^3^, matrix size = 400 × 400 × 400. Regardless of the TE, images (**a**–**j**) were obtained in 3D from asymmetric-echo radial spokes of the same lengths. Black arrows in (**k**–**m**) indicate locations with strong phase variation.

When the image to be reconstructed has actual phase variation at the time of imaging, magnitude-based projection can suffer from artifacts caused by the loss of phase interference information. This can be alleviated by employing complex projections, namely by retaining the phase in the back projections of *ts*FBP. This point is illustrated in Fig. 4, where the image phase was varied by varying the echo time in the presence of static field (B_0_) inhomogeneity. Here an ACR phantom was imaged at 3 T with a two-channel head volume coil at five different echo times (TE = 0.18 to 10 ms) in the presence of susceptibility-induced B_0_ inhomogeneity as well as channel-dependent RF (B_1_, both transmit and receive) phase. Images were reconstructed from the individual channels and then combined as the sum of the squares. The magnitude-only projection can be seen to suffer severe loss of image quality at TE > 5 ms, compared to the complex projection. The locations of image blurring and signal drop-out in Fig. 4(d,e) corresponded to the locations of strong B_0_ inhomogeneity on the perimeter and near an air bubble, indicated by the arrows in Fig. 4(k-m). Since B_0_-induced phase is proportional to TE while B_1_ phase is TE-independent, image phase at low TE is dominated by the latter. In our example, this phase varied relatively slowly in space (Fig. 4n,o) and did not appear to degrade the image quality of magnitude-only projections very much at low TE (< 5 ms).

## Discussion

### Computation time and channel combination

One of the reasons why *g*FFT is widely used for 3D radial-data reconstruction is that it takes much shorter processing time than *c*FBP. In this study, we examined another type of FBP method, *ts*FBP, and demonstrated that its computational requirement could be dramatically lower than *c*FBP, and even be comparable to *g*FFT in some practical situations (Fig. 1 and Table 1).

Among the factors that influence the computational efficiency, Fig. 1 highlighted three, namely the number of receiver channels (*N*_Ch_), the final matrix size (*N*), and the convolution-kernel width (*ω*). *ts*FBP showed much better overall computational efficiency than *c*FBP, which was more pronounced as *N*_Ch_ decreased or *N* increased. In contrast, when compared to *g*FFT, the computational efficiency of *ts*FBP tended to increase as *N*_Ch_ increased, *N* decreased, or *ω* increased (*ω* is only relevant to *g*FFT).

We note that in Eqs. (4–6) and Fig. 1, the signal combination for different receiver channels was assumed to be done before the back-projection for the FBP methods, while in *g*FFT the channel combination was done in the last step. It is possible for the FBP methods also to first create channel-specific images and then have them combined later. Such approach has several benefits. First, it would allow coil sensitivity profiles to be used for better signal-to-noise ratio (SNR) in the final image (Supplementary Fig. S5, demonstrating about 17% SNR difference). Second, coil-by-coil reconstruction better maintains *k*-space center consistency among different radial spokes, which can result in reduced intensity artifacts compared to projection-level coil combination (e.g., Fig. S5a compared to Fig. S5b). Finally, it could enable parallel imaging such as radial sensitivity encoding (SENSE) with iterative reconstruction^13^, provided complex projection is utilized. In such a (channel-wise reconstruction) case, the reconstruction time estimates Eqs. (4, 5) should be modified to have one factor of ${N}_{Ch}$ multiplying all the individual steps. ${N}_{Ch}$ will then be common to all three methods so the ratios between computational burden estimates will reduce to those with ${N}_{Ch}$ = 1 in Fig. 1. In this case, *ts*FBP is still more than 30 times faster than *c*FBP in our example (Fig. 1a), but its speed comparison with *g*FFT becomes less favorable (at most faster by factor of 2 and slower in most cases, Fig. 1d, ${N}_{Ch}$ = 1).

We also note that one can consider performing channel combination before gridding in *g*FFT. This would be possible in case of magnitude-based reconstruction, in which multiple receivers’ projection data are first combined by 1D FFT magnitude summation, and then interpolated to the Cartesian grid for 3D FFT. This will decrease the computation time for *g*FFT by approximately *N*_Ch_. The computational speed comparison will then again reduce to the case of *N*_Ch_ = 1 in Eqs. (4, 5) and Fig. 1.

Advanced computing hardware such as multiple CPU cores can substantially improve the computational speed independent of the reconstruction method. The fact that the proposed method, *ts*FBP, can also benefit from multi-core computation is illustrated in Table 2, where we listed measured image reconstruction times for the three methods obtained with 1, 3, 6 cores. We observed that the hardware-enabled acceleration was comparable between *ts*FBP and *g*FFT (an order of magnitude), while the gain was somewhat weaker for the slowest method, *c*FBP.

**Table 2:** Comparison of actual computation times for the 3 T experiment, according to the number of cores for parallel computation.

Methods	Actual computation times		
	1-Core	3-Core	6-Core
Two-step 2D FBP	1 min 30 s	25 s	10 s
3D FFT via gridding	10 min	2 min 50 s	1 min
Conventional 3D	380 min	140 min	90 min

### Magnitude versus complex projections

*ts*FBP with magnitude-only projection has certain advantages over *g*FFT in terms of the tolerance to off-centering errors of *k*-space trajectories that might result from gradient timing delays, eddy currents, or finite digitization bandwidth. In *g*FFT that is phase sensitive, off-centered *k*-space data creates unwanted phase modulation in the image space, causing contrast-varying artifacts. Several approaches have been proposed to mitigate the *k*-space off-centering problem for *g*FFT reconstruction. For example, the gradient delay could be compensated by realigning the radial data in post-processing with another calibration scan^14^ or by monitoring the actual gradient strength during the scan to estimate the actual *k*-space trajectory^15^. These and most other corrections require additional scans or hardware, and do not guarantee complete removal of off-centering artifacts when the *true* echo peak is placed between two adjacent samples, which is inherently possible in discrete sampling with a finite sampling bandwidth (Fig. 3). While simple 1D interpolation of the radial data can effectively increase the sampling frequency and reduce the digitization error in *g*FFT, this will increase the grid size and therefore the overall processing time. In comparison, magnitude-based FBP is inherently more robust against trajectory errors as long as they are purely radial. Obviously, however, loss of phase information in magnitude-only method precludes applications such as phase-based susceptibility imaging^16,17^ and phase-contrast flow imaging^18–20^.

As was shown in Fig. 4, when significant phase variation exists in the image, such as due to B_0_ inhomogeneity^21^ or RF phase, magnitude-only FBP will fail to reconstruct the true image magnitude because of the unrecoverable, destructive interference in 1D projections. This drawback will be more severe for higher magnetic fields and longer echo times, larger field of view (FOV), and more localized RF coils. Such problems can be mitigated by employing complex projections (Fig. 4).

### *k*-space trajectory

Another limitation of the proposed *ts*FBP method comes from the requirement of a specific radial spoke placement which is not spherically symmetric. An immediate consequence of such asymmetry is that the point spread function is anisotropic as illustrated in Supplementary Fig. S6. Furthermore, in order to satisfy the Nyquist criterion to avoid under-sampling artifacts, the spokes on the equator should satisfy (we use definition of *k* without 2$\uppi$)

$${k}_{max}\Delta \phi ={k}_{max}\Delta \theta =1/FOV$$

where the maximum *k*-space extent is given by $${k}_{max}=\left(1/2\right) N/FOV$$ assuming an isotropic field of view and grid size ($${N}_{x}={N}_{y}={N}_{z}=N)$$. In *ts*FBP, the number of half-echo radial spokes satisfying the above criterion is

$${N}_{v,tsFBP}=\left(\frac{\pi }{\Delta \phi }\right)\left(\frac{2\pi }{\Delta \theta }\right)=2\cdot {\left(\frac{\pi }{2}N\right)}^{2}.$$

This compares with the required number of spokes in uniform spherical sampling,

$${N}_{v,unif}=\frac{4\pi }{\Delta \phi\Delta \theta }=4\pi {\left(\frac{N}{2}\right)}^{2}.$$

Therefore, ${N}_{v,tsFBP}$ is $\uppi /2\approx 1.57$ times larger than ${N}_{v,unif}$, which means that *ts*FBP takes 1.57 times longer acquisition than *g*FFT based on uniform radial sampling. When the number of spokes is short of the Nyquist condition, under-sampling artifacts appear in *ts*FBP as illustrated for specific cases in Supplementary Fig. S7. The artifact pattern depends on the angular direction in which under-sampling occurs. While the additional scan time by 1.57 for full Nyquist sampling is significant, in our experience *ts*FBP with reduced spokes, namely ${{N}_{v,unif}\le N}_{v}<{N}_{v,tsFBP}$, incurred relatively minor SNR penalty (2–3% in case of ${{N}_{v}= N}_{v, unif}$ , Supplementary Fig. S8). Theoretically, this is understood by the following formula to account for different trajectories in SNR calculation^22^:

$$\mathrm{SNR\, ratio}={\left(\frac{{\int }_{0}^{{k}_{max}}\left(1/{D}_{2}\left(k\right)\right){k}^{2}dk}{{\int }_{0}^{{k}_{max}}\left(1/{D}_{1}\left(k\right)\right){k}^{2}dk}\right)}^\frac{1}{2}.$$

With *D*_1_(*k*) and *D*_2_(*k*) replaced by the mean *k*-space density functions for *ts*FBP and uniform trajectories, we obtain the SNR ratio of 0.977, in close agreement with experimental observation.

## Conclusion

In conclusion, the *ts*FBP method can bring high computational efficiency to classical back projection reconstruction of 3D radial data. With magnitude-only projection, it is relatively insensitive to radial *k*-space off-centering errors. For phase-sensitive imaging or for iterative reconstruction, *ts*FBP can still be used with complex projection. We expect that *ts*FBP could be a useful addition to the existing image reconstruction toolsets for 3D radial scan MRI which is receiving more and more attention for short echo times as well as relative motion insensitivity.

## Methods

### Comparison of conventional 3D and 2D back-projection methods

For conventional filtered back-projection (*c*FBP), a set of planar projections are obtained using the 3D projection slice theorem and then back-projected onto the 3D image space for reconstruction. In this reconstruction process, every radial spoke data $s\left(k,\theta ,\phi \right)$ is back-projected with a filter of ${k}^{2}\mathrm{sin}\theta$ as in the following equation^23^:

1 $$I\left(x,y,z\right)=I\left(\overrightarrow{r}\right)={\int }_{0}^{2\uppi }d\phi {\int }_{0}^{\uppi }d\theta {\int }_{0}^{\infty }dk \ {k}^{2}\mathrm{sin}\theta \ s\left(k,\theta ,\phi \right){e}^{i2\pi \overrightarrow{k}\cdot \overrightarrow{r}}.$$

On the other hand, the two-step filtered back-projection (*ts*FBP) performs image reconstruction using 2D filtered back-projection in two steps in which a ramp filter $\left|k\right|$ is applied to each radial data $s\left(k,\theta \right)$, as in the following equation^23^:

2 $$I\left(x,y\right)=I\left(\overrightarrow{r}\right)={\int }_{0}^{\pi }d\theta {\int }_{-\infty }^{\infty }dk \left|k\right| s\left(k,\theta \right) {e}^{i2\pi \overrightarrow{k}\cdot \overrightarrow{r}}.$$

Equations (1–2) are generally valid when the image to be reconstructed has spatially dependent phase across it caused by static field inhomogeneity or radio frequency (RF) excitation or reception phase. When such phase variation can be neglected, for example thanks to a very short echo time or relatively uniform RF fields, then the k-space function $s$ is Hermitian symmetric, and the integral along a full radial line in *k*-space will be real (up to a constant phase). In such a case, filtered back-projection can be done with magnitude projections only. For example, in 2D, Eq. (2) can be replaced by:

3 $$I\left(x,y\right)=I\left(\overrightarrow{r}\right)={\int }_{0}^{\pi }d\theta \left|{\int }_{-\infty }^{\infty }dk \left|k\right| s\left(k,\theta \right) {e}^{i2\pi \overrightarrow{k}\cdot \overrightarrow{r}}\right|.$$

Utilizing ultra-short echo-time (UTE) imaging and single- or few-channel volumetric RF coils, we experimentally observed that magnitude projections often yielded acceptable image quality.

### Two-step 2D filtered back-projection (*ts*FBP) method

**Figure 5:**
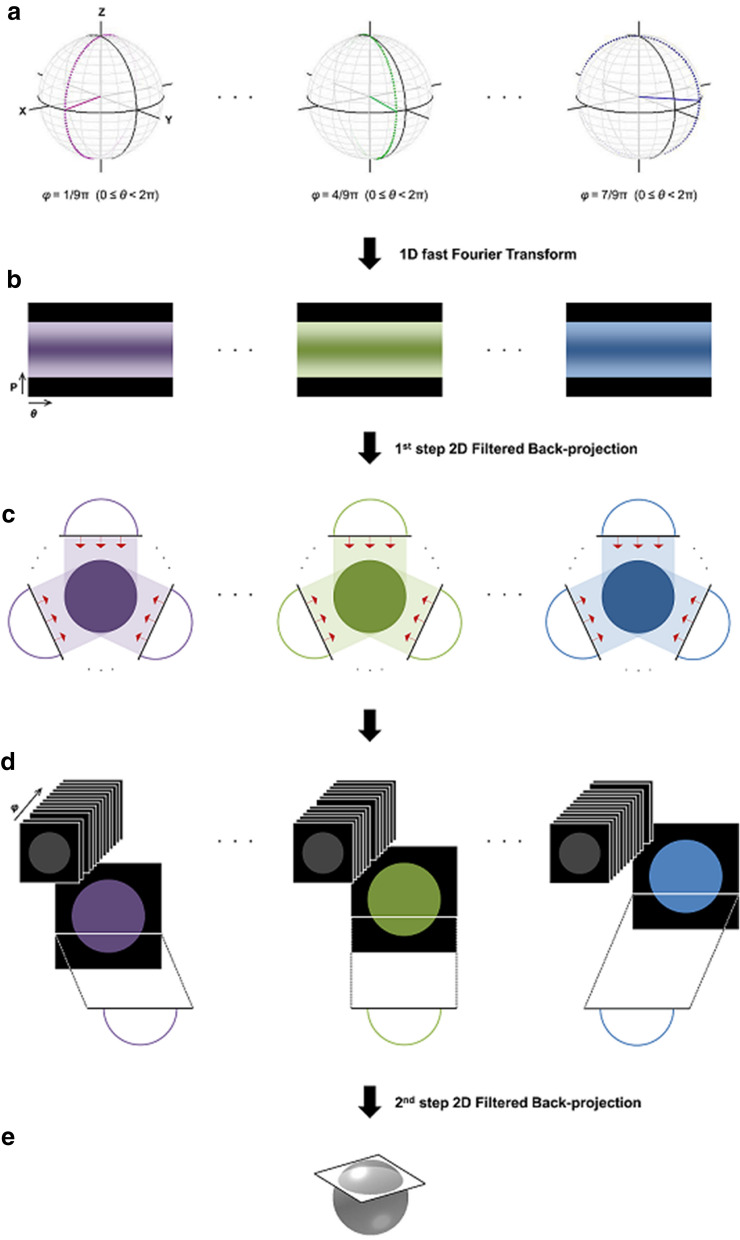
3D radial-data acquisition scheme and reconstruction flow chart of two-step 2D filtered back-projection. (**a**) Radial data is acquired in a series of 2D radial spokes filling the *k*-space in the form of vertical discs by incrementing the polar angle *θ* for each azimuthal angle *φ*. (**b**) One-dimensional fast Fourier transforms are performed to acquire a sinogram consisting of a series of 1D projections for each *φ*. (**c**) Two-dimensional projection images are reconstructed from each sinogram using the 1^st^-step 2D FBP for every *φ*. (**d**,**e**) For the 2^nd^-step 2D FBP, z-coordinate-matched strips taken from the stack of *φ*-dependent 2D projection images are 2D back-projected to form an image at each z.

The schematic diagram of the *ts*FBP method, including its unique data-acquisition scheme, is illustrated in Fig. 5. In *ts*FBP, 3D radial data are collected in a series of 2D radial spoke sets, each of which fills the *k*-space in the form of a vertical disc by incrementing the polar angle *θ* from 0 ≤ *θ* < π (full echo sampling) or 0 ≤ *θ* < 2π (free induction decay or partial echo sampling) for every azimuthal angle *φ* in the range of 0 ≤ *φ* < π (Fig. 5a). A specific reconstruction algorithm (outlined below) for this particular scheme of data collection is then applied to accomplish 3D image reconstruction. Note that while we used an ultra-short echo-time (UTE) sequence for radial data collection, in principle non-UTE radial acquisition is also compatible with *ts*FBP. In the presence of static field inhomogeneity, however, non-UTE acquisition would require complex projections to properly handle the image phase.

The 3D image reconstruction is achieved by the following steps. First, a sinogram consisting of a series of 1D projections is obtained from 1D FFT of all of the radial spokes on a vertical disc at a given *φ* in the *k*-space (Fig. 5b). Subsequently, 2D projection images are reconstructed from each sinogram using 2D FBP for every *φ* (1^st^-step 2D FBP; Fig. 5c). Finally, the *z*-coordinate-matched strips taken from the stack of *φ*-dependent 2D projection images are 2D back-projected to reconstruct a final 3D image slice by slice (2^nd^-step 2D FBP; Fig. 5d,e). For each step of *ts*FBP, a ramp-filter is applied prior to the back-projection.

### Mathematical expressions for computational requirement of each reconstruction method

In order to evaluate the computational requirement of each reconstruction method independent of particular programming environment, we analyzed the number of major operations required for the reconstruction process.

Firstly, *ts*FBP can be divided into three main processes of 1D FFT, 1^st^-step 2D FBP, and 2^nd^-step 2D FBP. In this case, the number of operations for *ts*FBP is formulated as follows:

4 $${O}_{tsFBP}={N}_{v}\left({N}_{s}{log}_{2}\left({N}_{s}\right)\right){N}_{Ch}+\left({N}_{v,\theta }{N}_{x}{N}_{z}\right){N}_{v,\varphi }+\left({N}_{v,\varphi }{N}_{x}{N}_{y}\right){N}_{z},$$

where *N*_v_ (= *N*_v,θ_*N*_v,φ_) is the number of the acquired radial spokes; *N*_s_ is the number of sampling points per spoke; *N*_Ch_ denotes the number of receiver channels; *N*_x_, *N*_y_, *N*_z_ are the final matrix sizes in the *x*, *y*, *z* axes; *N*_v,θ_ is the number of *θ* increments in a vertical disc of Fig. 5a; and *N*_v,φ_ is the number of *φ* increments. In the first term of Eq. (4), *N*_s_log_2_(*N*_s_) represents the number of operations required for 1D FFT of each spoke. Multiplying that by the number of spokes (*N*_v_) and the receiver channels (*N*_ch_) yields the total number of operations needed for 1D FFT of all radial spokes. In the second term, which corresponds to the 1^st^-step 2D FBP process (Fig. 5c), *N*_v,θ_*N*_x_*N*_z_ represents the number of operations required for 2D FBP of 2D radial spokes at a given *φ*. Here, each of the line projections (*N*_v,θ_) is back-projected to a matrix of size *N*_x_*N*_z_. Its multiplication by the number of *φ* increments (*N*_v,φ_) then gives the total number of operations during the 1^st^-step 2D FBP process. The final, 2^nd^-step FBP term, which is similar to the 1^st^-step FBP, is obtained by multiplying the operational counts (*N*_v,φ_*N*_x_*N*_y_) for the 2D FBP of a *z*-coordinate-matched slice by the number of these slices (*N*_z_).

*c*FBP mainly consists of 1D FFT and 3D FBP, and the number of total operations is given by:

5 $${O}_{cFBP}={N}_{v}\left({N}_{s}{log}_{2}\left({N}_{s}\right)\right){N}_{Ch}+\left({N}_{x}{N}_{y}{N}_{z}\right){N}_{v}.$$

Here, the first term for the 1D FFT process is just the same as that of Eq. (4). In the second term, *N*_x_*N*_y_*N*_z_ represents the number of operations for the 3D back-projection performed at an angle of each spoke, and multiplication by the number of spokes (*N*_v_) gives the total number of 3D FBP operations.

For *g*FFT, the main steps consist of gridding, 3D FFT, and intensity correction. The number of operations in total is:

6 $${O}_{gFFT}=\left[{N}_{v}{N}_{s}{\omega }^{3}+{V}^{3}{N}_{x}{N}_{y}{N}_{z}{log}_{2}\left({V}^{3}{N}_{x}{N}_{y}{N}_{z}\right)+ {N}_{x}{N}_{y}{N}_{z}\right] {N}_{Ch}$$

where *ω* is the convolution-kernel width for gridding and *V* is the *k*-space oversampling factor. The first term corresponds to the 3D gridding operation for all the sampling points of the radial *k*-space data, whose number is given by the product of the acquired radial spokes and the sampling points per spoke (*N*_v_*N*_s_). The gridding algorithm distributes each sampling point to the Cartesian grid by convolving with a gridding kernel such as the Kaiser–Bessel window. The second term relates to the processing time of 3D FFT with an oversampling factor^24^. The final, intensity correction term captures the division process by the inverse Fourier transform of the convolution kernel at each voxel of the final image, which is intended to compensate for the interpolation effect due to the convolution-kernel application. That is multiplied by the number of channels (*N*_Ch_) since reconstruction is performed separately for each channel. Table 1 summarizes the mathematical expressions for the number of main operations at individual reconstruction stages of the three reconstruction methods.

It is worth mentioning at this point that *g*FFT can also be done with two-step 2D gridding, rather than 3D gridding, provided that the *k*-space trajectory is compatible with *ts*FBP as explained above. While such two-step *g*FFT was not part of our experimental tests, for completeness we list below the mathematical expression for its operation count.

7 $${O}_{tsgFFT}=\left[{N}_{v,\theta }{N}_{v,\phi }{N}_{s}{\omega }^{2}+{N}_{x}{N}_{v,\phi }{N}_{z}{\omega }^{2}+{V}^{3}{N}_{x}{N}_{y}{N}_{z}{log}_{2}\left({V}^{3}{N}_{x}{N}_{y}{N}_{z}\right)+ {{N}_{x}{N}_{z}{N}_{v,\phi }+N}_{x}{N}_{y}{N}_{z}\right] {N}_{Ch}.$$

Compared to Eq. (6), the above expression shows that the gridding and the intensity correction operations now each consist of two steps. For gridding (the first two terms), the interpolation kernel size is reduced from ${\omega }^{3}$ to ${\omega }^{2}$, reflecting 2D gridding. Each of the two gridding operations has roughly the same magnitude as the 3D gridding operation (${N}_{v}{N}_{s}{\omega }^{3}$ in Eq. 6). Therefore, Eq. (7) is comparable to Eq. (6) when $\omega =2$, but two-step *g*FFT will be more favorable for a large interpolation kernel size.

### Simulations

To compare the computational requirements, the number of major operations was calculated for each reconstruction method using Eqs. (4–6) as a function of an isotropic matrix size *N* = *N*_x_ = *N*_y_ = *N*_z_ with a varying number of receiver channels *N*_Ch_. Parameter setups for the simulation were: *N*_s_ = *N/2*, *N*_v_ = 2 (π *N*_s_)^2^ to satisfy the Nyquist criterion, *V* = 2, and *ω* = 2–4^25,26^. Values of the oversampling factor (*V*) and the convolution-kernel width (*ω*) were chosen to be typical for conventional *g*FFT reconstructions. Four simulations were carried out for comparison between *ts*FBP and *c*FBP as well as between *ts*FBP and *g*FFT, varying *ω* from two to four (Fig. 1).

Supplementary Fig. S7 shows images reconstructed by *ts*FBP while varying the number of polar (θ) and azimuthal (φ) angles. For this, we performed simulations using a 3D Shepp-Logan phantom. 3D radial signals corresponding to a set of polar (θ) and azimuthal angles (φ) were generated. The phantom grid size and the final reconstruction matrix size were both 128 × 128 × 128.

The basic accuracy of *ts*FBP reconstruction was verified against conventional methods through simulations on the same numerical phantom. Supplementary Figure S9 shows images reconstructed by *g*FFT, *c*FBP and *ts*FBP with different filter and interpolation functions used in the back-projection methods (*c*FBP, *ts*FBP). The following simulation parameters were used: (*ts*FBP)${N}_{v,\theta }$ = 201, ${N}_{v,\phi }$ = 201; (*c*FBP) *N*_*v*_ = 40,401; and (*g*FFT) *N*_*v*_ = 40,401,* V* = 2 and* ω = *4***.*** The difference images with respect to the ground truth showed that while *g*FFT was the most accurate, *ts*FBP performed better than or similarly to *c*FBP for the simulation parameters used.

### Experiments

In order to evaluate the computation times of each reconstruction method on actual scan data and also their robustness against off-centering of the *k*-space trajectory, 3D radial-scan experiments were performed on phantoms and the human brain on preclinical 9.4 T (BioSpec 94/30, Bruker, Ettlingen, Germany) and clinical 3 T (Magnetom Trio, Siemens, Erlangen, Germany) scanners. For the human brain imaging at 3 T, informed written consent was obtained from a healthy volunteer according to a protocol approved by the Sungkyunkwan University ethics committee. All data sets were obtained using the CODE (Concurrent Dephasing and Excitation) pulse sequence^27^, which is a 3D radial gradient-echo-based UTE sequence. The sequence generates a partial gradient echo for each radial spoke, sufficient to construct a 1D projection in the corresponding direction. We note that magnitude projections based on partial echoes can suffer from blurring and background brightening due to lost *k*-space data on the short-tail side. While this can be mitigated by homodyne filtering or more symmetric echoes, the CODE data were sufficient for our main purpose of computation time comparison and echo shift simulation. All the 1D projection data for radial reconstruction were obtained such that the end points of the corresponding radial spokes spanned the whole (not half) *k*-space sphere.

First, the standard ACR phantom and the human brain scans were performed at 3 T using a four-channel head coil. Scan parameters were: *N*_v_ = 64,800, *N*_v,φ_ = 180, *N*_v,θ_ = 360, TR = 3 ms, TE = 0.22 ms, flip angle = 5°, FOV = 300 × 300 × 300 mm^3^, and spatial resolution = 1 × 1 × 1 mm^3^. Second, another phantom imaging was also performed at 9.4 T using a single-channel volume coil. Scan parameters were: *N*_v_ = 64,800, *N*_v,φ_ = 180, *N*_v,θ_ = 360, TR = 3 ms, TE = 0.15 ms, flip angle = 5°, FOV = 60 × 60 × 60 mm^3^ , and spatial resolution = 0.47 × 0.47 × 0.47 mm^3^.

Data obtained at 3 T and 9.4 T were also used to show the robustness of *ts*FBP to the off-centering errors of the *k*-space trajectories in comparison to *g*FFT. For better demonstration of the *k*-space off-centering effects and tolerance to them, some echo peaks of the 3 T scan data were manually shifted during the reconstruction process. In other words, while the echo peaks were located between the 3rd and 6th points in the acquired data (“as-acquired data”), some of the echo peaks were randomly shifted by up to 15 points from their original locations to create a larger deviation of the echo-peak positions (for detailed information of the echo-peak distributions, refer to Fig. S1 in Supplementary Information).

In addition, another source of mis-registration of the center of *k*-space (digitization error) was investigated using the phantom data at 9.4 T. Due to the finite digitization bandwidth, sometimes the echo-peak position of a discretely sampled echo signal does not exactly coincide with the true *k* = 0 even after correction of the shifts induced by gradient delays and eddy currents. That is, the true *k* = 0 is placed between two sampling points. It was shown that, in this case, the image quality can be further improved by a manual echo shift smaller than the sampling interval for *g*FFT (Fig. 3).

### Image reconstruction

The three reconstruction methods of *g*FFT, *c*FBP, and *ts*FBP were applied to image reconstruction of 3D radially sampled CODE data, and their computation times were recorded during the reconstruction processes. All calculations were carried out offline using Matlab (R2011a, MathWorks, Natick, MA, USA) on a workstation equipped with an Intel-Xeon CPU (3.50 GHz). We note that other available packages such as Berkeley Advanced Reconstruction Toolkit (BART)^28^ could be used for reconstruction and could take different computation times. Given the generally good agreement between the mathematical operation counts and our Matlab results, however, we do not believe that using other packages will significantly change our conclusion. For the *g*FFT reconstruction, the mean value of all echo-peak positions was used to define *k* = 0. The convolution-kernel width (*ω*) was set as two.

## Supplementary information

Supplementary file1.

## Data Availability

The datasets generated and analyzed during the current study are available from the corresponding author upon reasonable request. An example to compare the image reconstruction methods proposed in this study is available online. To use example related data and code, please use the link below (https://drive.google.com/drive/folders/12U1ERpsoM2G7Ku5Nnc9drpllPMbixBs-?usp=sharing).
