# Supplementary material for: Strategies for rapid reconstruction in 3D MRI with radial data acquisition: 3D fast Fourier transform vs two-step 2D filtered back-projection
Source: Sci Rep. 2020 Aug 14;10:13813. doi: 10.1038/s41598-020-70698-4 (PMC7427795; doi:10.1038/s41598-020-70698-4)

**Supplementary Information**

**Strategies for Rapid Reconstruction in 3D MRI with Radial Data Acquisition: 3D Fast Fourier Transform vs. Two-Step 2D Filtered Back-Projection**

*Jinil Park^1^, Jeongtaek Lee^1^, Joonyeol Lee^1,2^, Seung-Kyun Lee^1,2^, Jang-Yeon Park^1,2^*

1. Department of Biomedical Engineering, Sungkyunkwan University, Suwon, South Korea

2. Center for Neuroscience Imaging Research, Institute for Basic Science (IBS), Suwon, South Korea


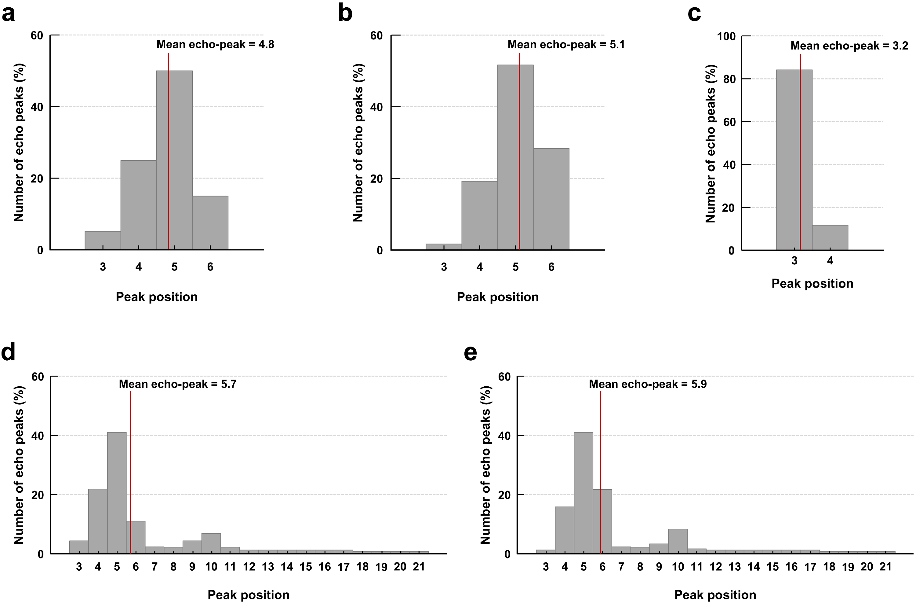


**Supplementary Fig. S1.** Histograms of the echo-peak positions. The mean echo-peak position is indicated by a red solid line in each histogram. **(a-c)** Echo-peak distributions of the as-acquired data from the ACR phantom (a), the human brain at 3T (b), and a home-built phantom at 9.4T (c). (**d-e)** Histograms of the echo-peak positions after manually shifting some of the peaks in **a** (d), and **b** (e).


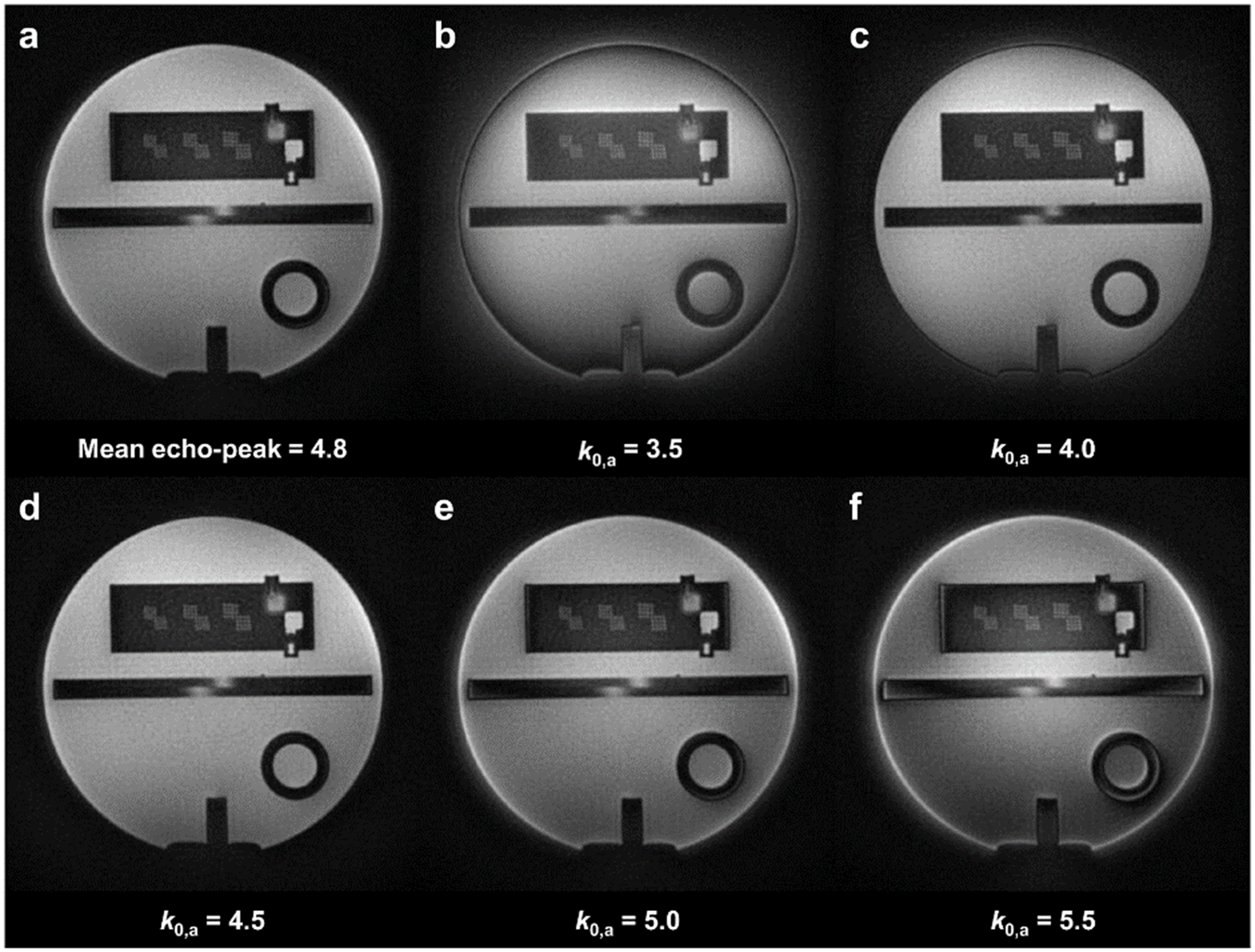


**Supplementary Fig. S2.** ACR phantom images at 3T reconstructed with *g*FFT, varying the position of the *apparent* *k* = 0 (*k*_0,a_). **(a)** The image was reconstructed using the mean echo-peak position (4.8) for *k* = 0. **(b-f)** The images were reconstructed after shifting *k*_0,a_ from 3.5 to 5.5 sample points by an increment of 0.5 point. Image (d) shows better image quality than the others, implying that its position of *k*_0,a_, i.e., 4.5 sample points, is the closest to the *true k =* 0.


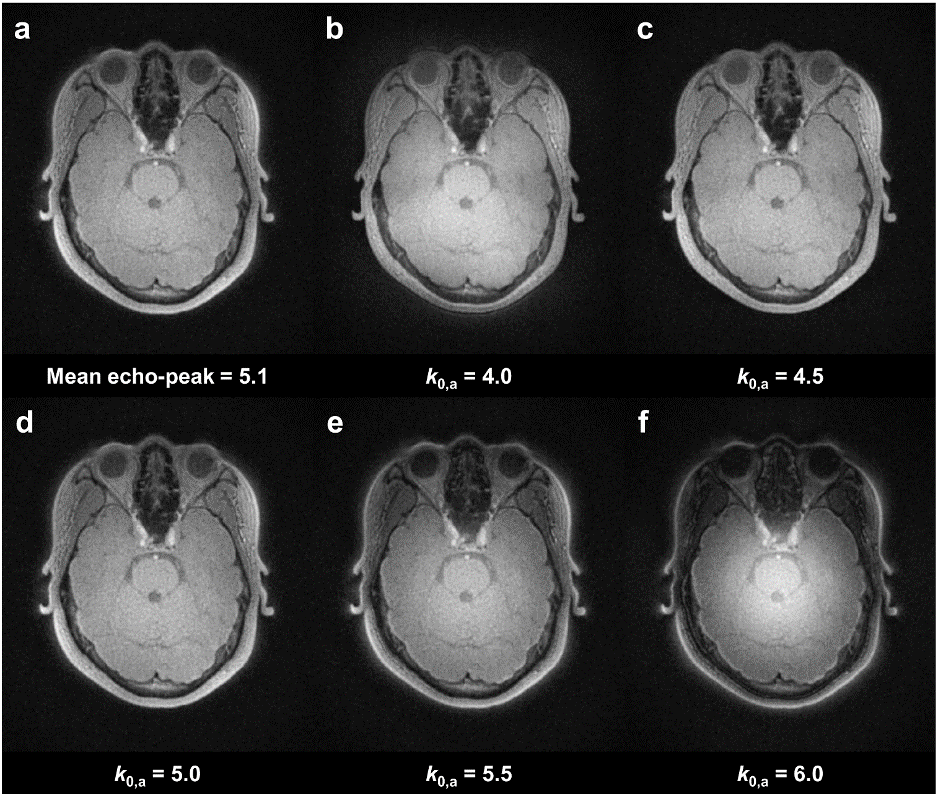


**Supplementary Fig. S3.** Human brain images at 3T reconstructed with *g*FFT, varying the position of the *apparent* *k* = 0 (*k*_0,a_). **(a)** The image was reconstructed using the mean echo-peak position (5.1) for *k* = 0. **(b-f)** The images were reconstructed after shifting *k*_0,a_ from 4.0 to 6.0 sample points by an increment of 0.5. Images (a) and (d) show similar, less shaded image qualities, implying that their positions of *k*_0,a_ are closer to the *true* *k* = 0 than those of the others.


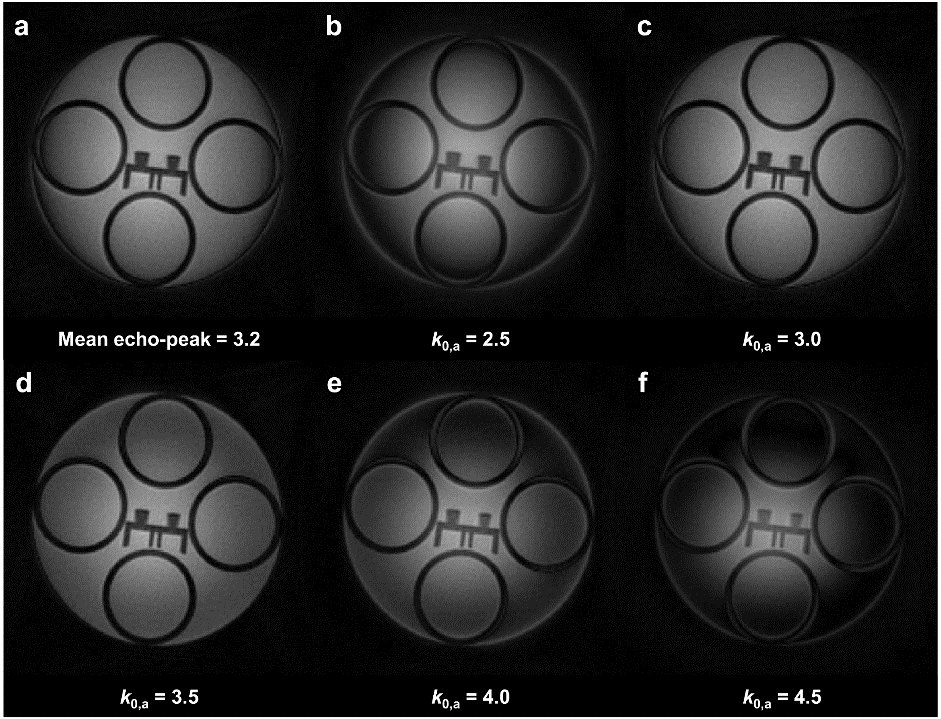


**Supplementary Fig. S4.** Home-built phantom images at 9.4T reconstructed with *g*FFT, varying the position of the *apparent* *k* = 0 (*k*_0,a_). **(a)** The image was reconstructed using the mean echo-peak position (3.2) for *k* = 0. (**b**-**f)** The images were reconstructed after shifting *k*_0,a_ from 2.5 to 4.5 sample points by an increment of 0.5. Image (d) shows the best image quality in terms of edge artifacts, implying that its position of *k*_0,a_ is closer to the *true* *k* = 0 than the others.

**
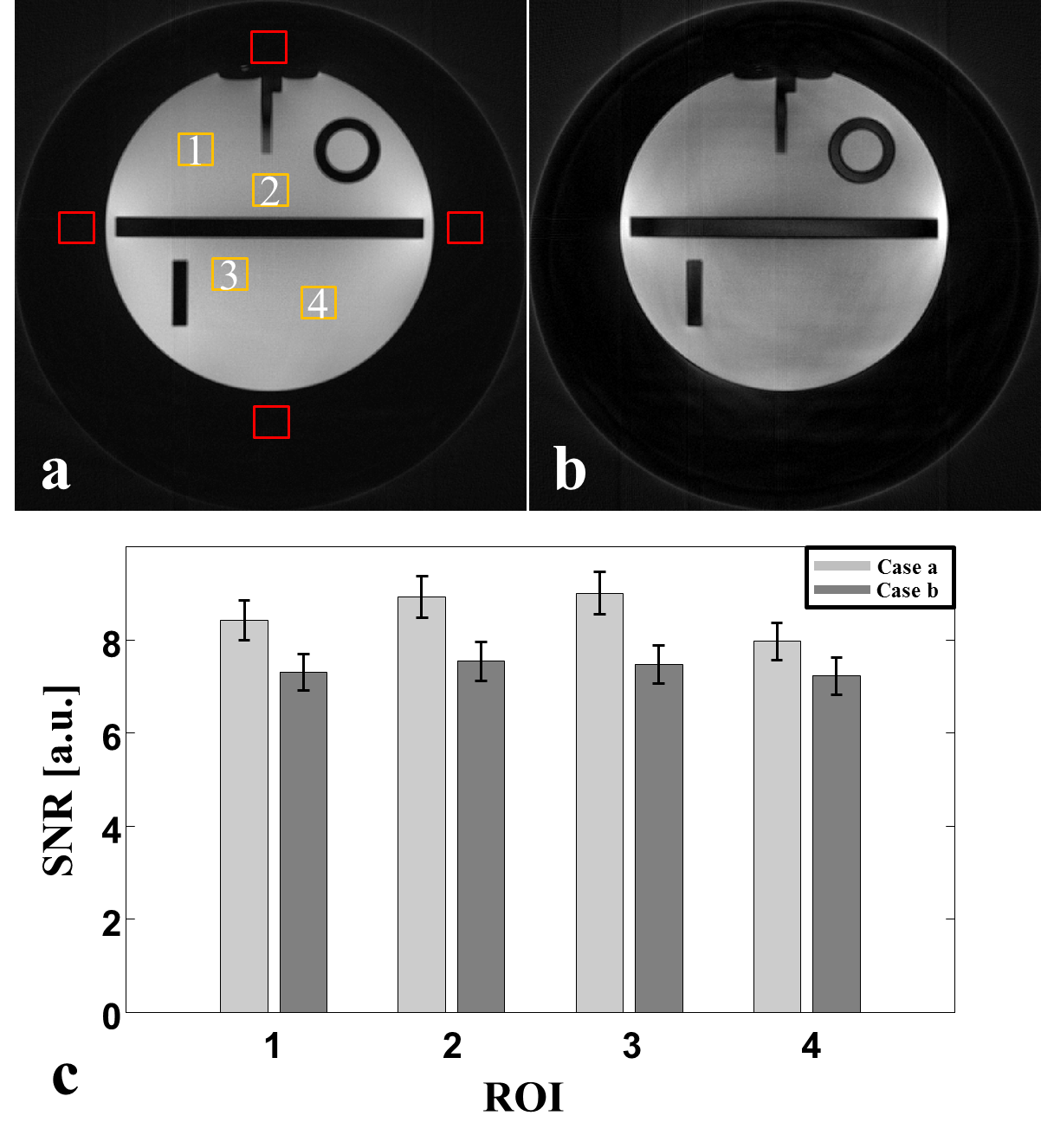
**

**Supplementary Fig. S5.**

Images reconstructed by combining individual channel images (a), and by combining the 1D projection magnitudes from different channels prior to full reconstruction (b) in *ts*FBP. Yellow and red boxes indicate the ROIs for signal and background noise measurements, respectively. SNR comparison in the ROIs shows up to 17% decrease of SNR in case (b). Scans were performed at 3T with a 20-channel head coil. The *ts*FBP trajectory and reconstruction parameters were: *N_v,θ_* = *N_v,φ_* = 360, *Ns* = 250, *N* = 500.

**Supplementary Fig. S6.** K-space sampling distributions and corresponding point spread functions for different radial sampling patterns including a uniform shell trajectory (a) and the *ts*FBP trajectories (b-k) with different spoke numbers. The image matrix size *N* = 128 was used for which the number of (full-echo) radial spokes satisfying the Nyquist criterion is 25,736 for the shell trajectory and 40,401 for *ts*FBP. **(a-b)** Fully sampled cases. **(c-e)** Point spread functions when *ts*FBP is under-sampled in the azimuthal ($\phi$) direction. **(f-h)** Point spread functions when under-sampling is in the polar ($\theta$) direction. **(i-k)** Point spread functions for under-sampling in both $\phi$ and $\theta$.

**
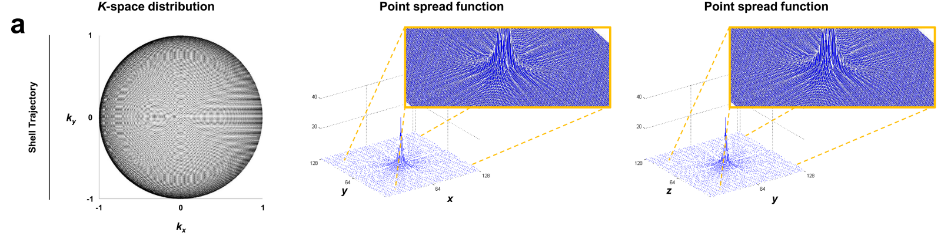

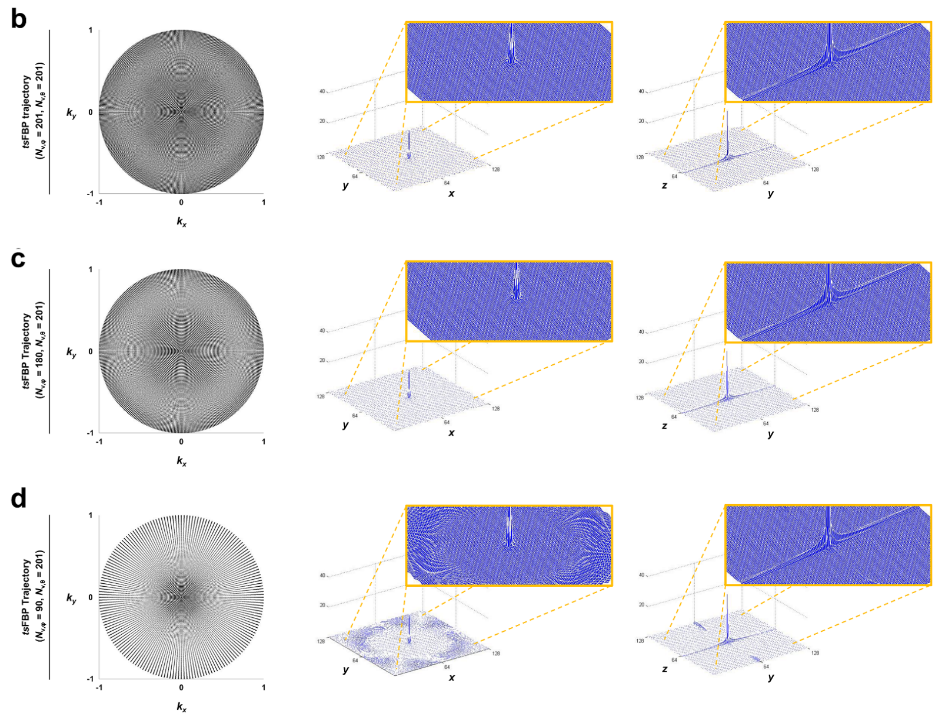
**

**
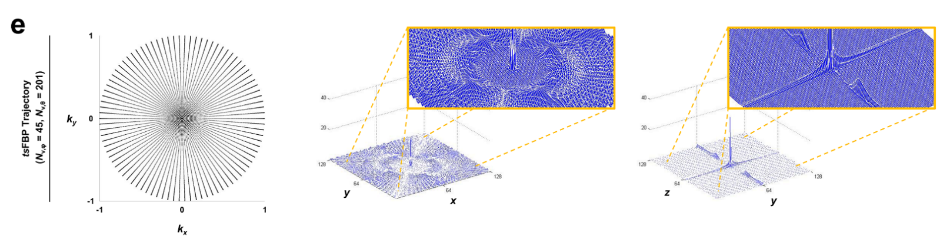
**

**
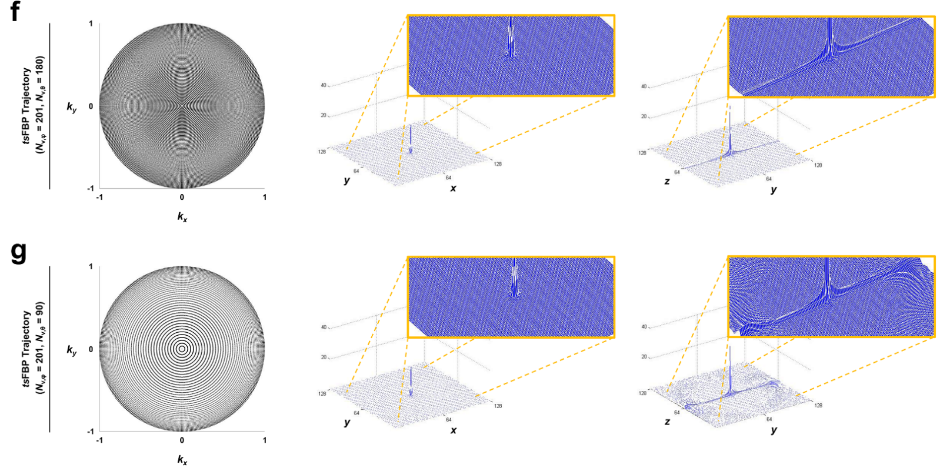
**

**
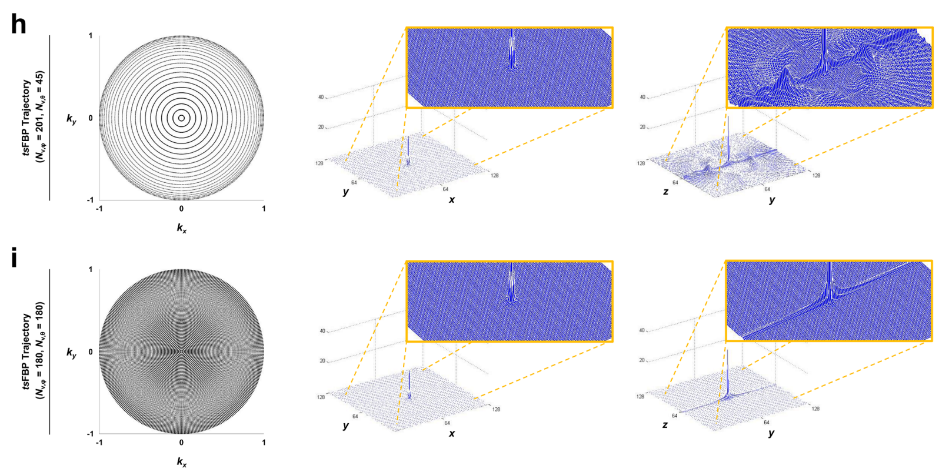
**

**
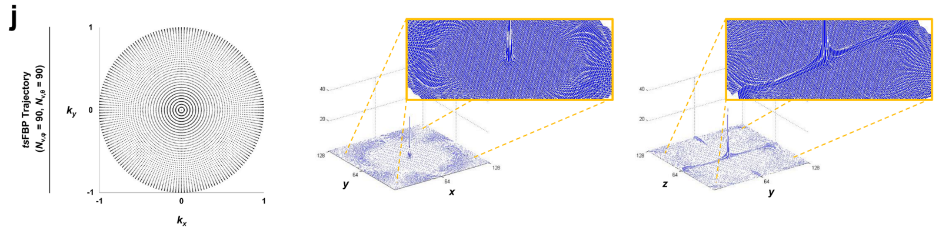
**

**
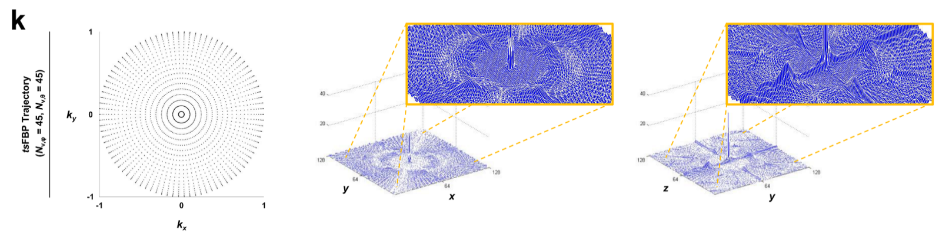
**

**Supplementary Fig. S7.** Simulated 3D Shepp-Logan phantom images reconstructed by *ts*FBP varying the numbers of polar and azimuthal angles. The phantom FOV was 128 × 128 × 128 for which the full (Nyquist) sampling requires $N_{v,\theta}\times N_{v,\phi}=201\times201$ radial spokes. Under-sampling artifacts appear as the number of the spokes is reduced.


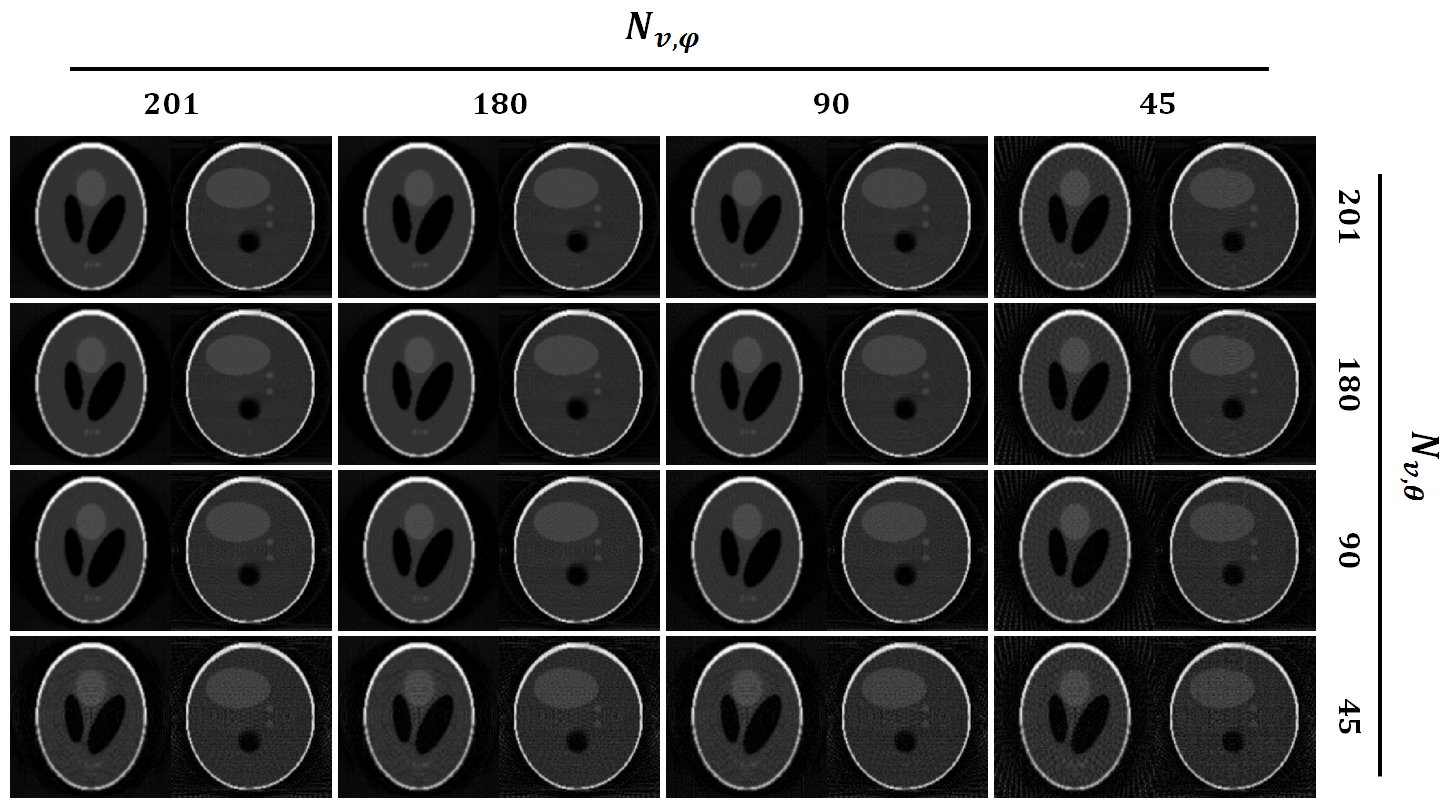


**Supplementary Fig. S8**. SNR comparison for the same number of radial spokes. Images were reconstructed from a uniform shell trajectory (a) and *ts*FBP trajectories with two different combinations of *N*_v,ϕ_ and *N*_v,θ_ (b, c) with identical total spokes count (51,360). SNR comparison (d) shows relatively minor differences. Yellow and red boxes in (a) indicate the ROIs for signal and noise measurements, respectively. The scan was performed on a cylindrical water phantom at 9.4T using a volume coil, with parameters: TR = 4 ms, TE = 8 us, FA = 5°, FOV = 60 × 60 × 60 mm^3^, matrix size = 128 × 128 × 128. The number of the center-out radial spokes used (51,360) very nearly satisfied the Nyquist condition for the uniform shell trajectory.


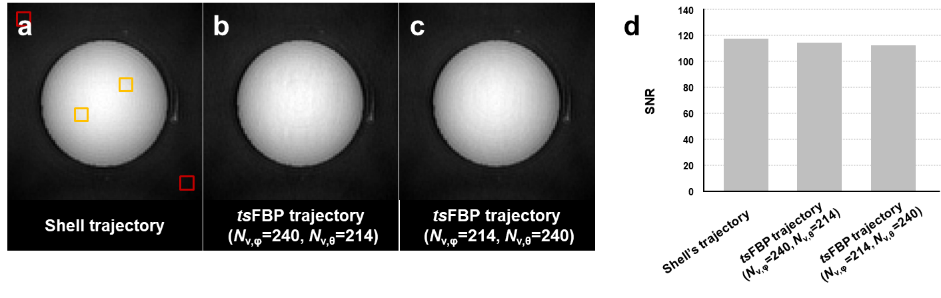


**Supplementary Fig. S9.** Representative axial slices of 3D Shepp-Logan phantom (a) and reconstructed images with *c*FBP (b-d), *g*FFT (e), and *ts*FBP (f-h). The three *c*FBP and *ts*FBP images used different combinations of the filters and interpolations: From left to right, Ram-Lak filter/linear interpolation (2^nd^ column, used in the rest of the paper); Ram-Lak filter/nearest neighbor interpolation (3^rd^ column); Hann filter/linear interpolation (4^th^ column). The difference images for all reconstruction methods with respect to the reference (ground truth, a) are shown in (i-o) with ×10 magnified grayscale.


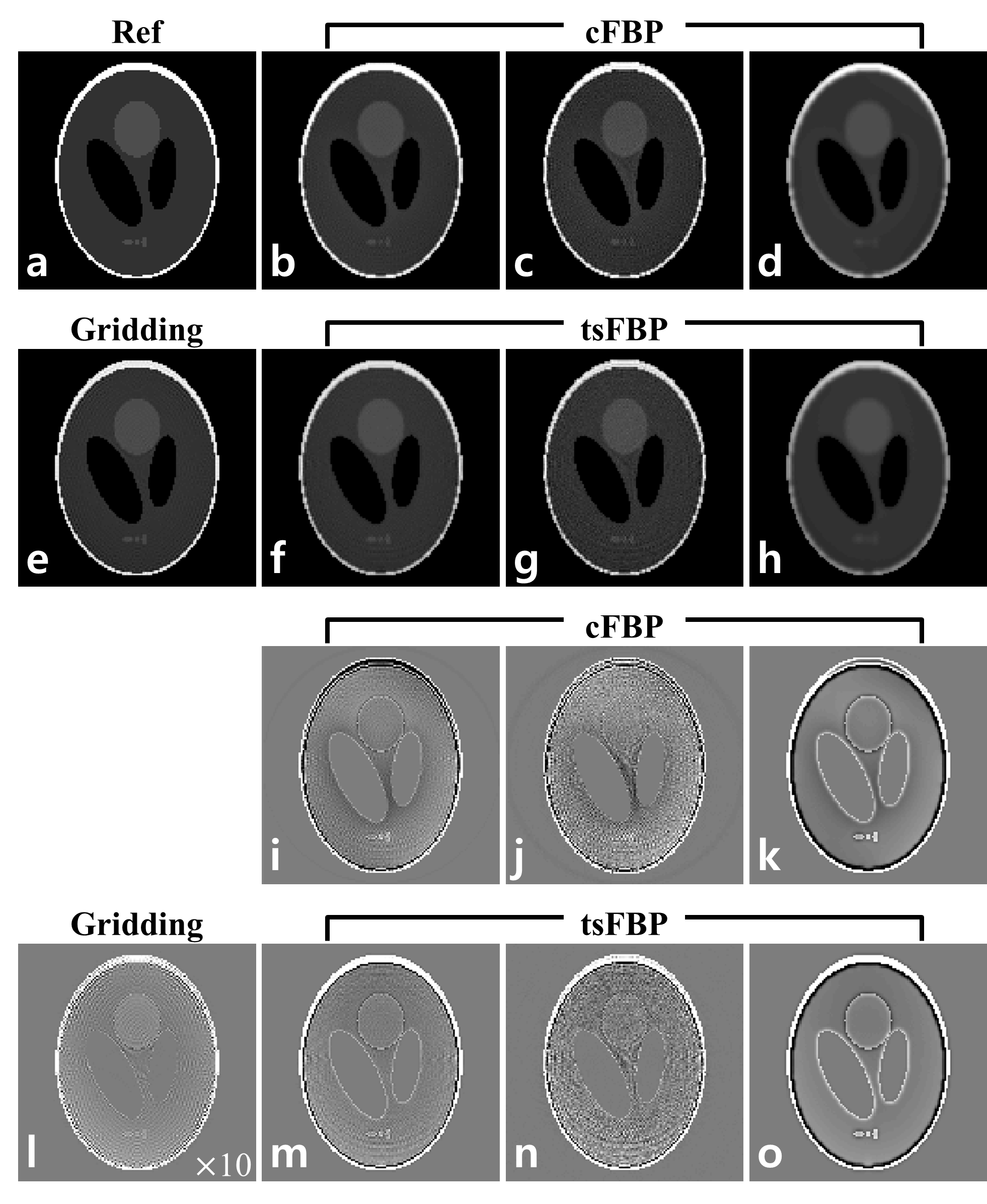

Supplement: Supplementary file 1 — Supplementary file1. [file 41598_2020_70698_MOESM1_ESM.docx]
